# Phase II study of trifluridine/tipiracil plus bevacizumab by *RAS* mutation status in patients with metastatic colorectal cancer refractory to standard therapies: JFMC51-1702-C7

**DOI:** 10.1016/j.esmoop.2021.100093

**Published:** 2021-03-19

**Authors:** T. Takahashi, K. Yamazaki, E. Oki, M. Shiozawa, K. Mitsugi, A. Makiyama, M. Nakamura, H. Ojima, Y. Kagawa, N. Matsuhashi, H. Okuda, M. Asayama, Y. Yuasa, Y. Shimada, D. Manaka, J. Watanabe, K. Oba, T. Yoshino, K. Yoshida, Y. Maehara

**Affiliations:** 1 Department of Digestive Surgery, Gifu University Hospital, Gifu, Japan; 2 Division of Gastrointestinal Oncology, Shizuoka Cancer Center, Shizuoka, Japan; 3 Department of Surgery and Science, Kyushu University, Fukuoka, Japan; 4 Department of Gastrointestinal Surgery, Kanagawa Cancer Center, Kanagawa, Japan; 5 Department of Medical Oncology, Hamanomachi Hospital, Fukuoka, Japan; 6 Department of Hematology/Oncology, Japan Community Healthcare Organization Kyushu Hospital, Fukuoka, Japan; 7 Department of Gastroenterology, Sapporo City General Hospital, Hokkaido, Japan; 8 Department of Gastroenterological Surgery, Gunma Prefectural Cancer Center, Gunma, Japan; 9 Department of Surgery, Kansai Rosai Hospital, Hyogo, Japan; 10 Department of Medical Oncology, Keiyukai Sapporo Hospital, Hokkaido, Japan; 11 Department of Gastroenterology, Saitama Cancer Center, Saitama, Japan; 12 Department of Gastrointestinal Surgery, Tokushima Red Cross Hospital, Tokushima, Japan; 13 Division of Clinical Oncology, Kochi Health Sciences Center, Kochi, Japan; 14 Department of Surgery, Kyoto Katsura Hospital, Kyoto, Japan; 15 Department of Surgery, Gastroenterological Center, Yokohama City University Medical Center, Kanagawa, Japan; 16 Department of Interfaculty and Initiatives in Information Studies, University of Tokyo, Tokyo, Japan; 17 Department of Gastroenterology and Gastrointestinal Oncology, National Cancer Center Hospital East, Chiba, Japan; 18 Kyushu Central Hospital of the Mutual Aid Association of Public School Teachers, Fukuoka, Japan

**Keywords:** metastatic colorectal cancer, bevacizumab, trifluridine/tipiracil, RAS mutation status, JFMC51-1702-C7 trial

## Abstract

**Background:**

Although the efficacy of trifluridine/tipiracil (FTD/TPI) plus bevacizumab (BEV) against metastatic colorectal cancer (mCRC) has been demonstrated, little is known about its effectiveness upon disease stratification by *RAS* mutations. In this phase II study, we investigated the efficacy and safety profiles of FTD/TPI in mCRC according to *RAS* mutation status.

**Patients and methods:**

Eligible patients were mCRC refractory or intolerant to all standard therapies other than FTD/TPI and regorafenib. Patients received 4-week cycles of treatment with FTD/TPI (35 mg/m^2^, twice daily, days 1-5 and 8-12) and bevacizumab (5 mg/kg, days 1 and 15). The primary endpoint was disease control rate (DCR). The null hypothesis of DCR in both *RAS* wild-type (WT) and mutant (MUT) cohorts was 44%, assuming a one-sided significance level of 5.0%. The necessary sample size was estimated to be 49 patients (target sample size: 50 patients) for each cohort.

**Results:**

Between January and September 2018, 102 patients were enrolled, and 97 patients fulfilled the eligibility criteria (48 in the *RAS* WT cohort and 49 in the *RAS* MUT cohort). DCRs in the *RAS* WT and MUT cohort were 66.7% [90% confidence interval (CI), 53.9%-77.8%, *P* = 0.0013] and 55.1% (90% CI, 42.4%-67.3%, *P* = 0.0780), respectively. The median progression-free survival (PFS) and overall survival (OS) were 3.8 and 9.3 months, respectively, in the *RAS* WT cohort and 3.5 and 8.4 months, respectively, in the *RAS* MUT cohort. The most common grade 3 or higher adverse event in both cohorts was neutropenia (46% in the *RAS* WT cohort and 62% in the *RAS* MUT cohort), without unexpected safety signals.

**Conclusions:**

FTD/TPI plus bevacizumab showed promising activity with an acceptable safety profile for pretreated mCRC, regardless of *RAS* mutation status, although the efficacy outcomes tended to be better in *RAS* WT.

## Highlights

• We investigated the efficacy and safety of FTD/TPI in mCRC according to *RAS* mutation status.
• DCRs in the *RAS* WT and MUT cohorts were 66.7% and 55.1%, respectively.
• PFS and OS did not differ significantly according to *RAS* mutation status.
• The most common adverse event (grade 3 or higher) in both cohorts was neutropenia.
• FTD/TPI plus BEV treatment showed promising activity for pretreated mCRC, regardless of *RAS* mutation status.

## Introduction

Colorectal cancer (CRC) is the third most common cancer and the second leading cause of cancer-related death worldwide.^1^ With advancements in treatment using combinations of cytotoxic drugs and molecular-targeted therapies for metastatic CRC (mCRC) in the past two decades, the median overall survival (OS) has reached approximately 30 months for selected patients in clinical trials.^2^, ^3^, ^4^, ^5^, ^6^ Recently, personalized treatments for mCRC have progressed owing to the development of treatments based on biomarkers, such as microsatellite instability (MSI) status and *BRAF* V600E mutation status, in addition to *RAS* mutation status.^7^, ^8^, ^9^

Trifluridine/tipiracil (FTD/TPI) is an oral antitumor drug that has been shown to significantly prolong survival in patients with mCRC. A phase III study, RECOURSE, showed that FTD/TPI exhibits superiority compared with placebo in terms of OS and progression-free survival (PFS) in patients with mCRC refractory to standard therapies.^10^ Another phase III study, TERRA, also showed consistent results in Asian patients with mCRC.^11^ Based on these results, FTD/TPI has been established as standard late-line therapy, according to several published guidelines.^12^, ^13^, ^14^, ^15^

Bevacizumab (BEV) enhances efficacy in combination with standard chemotherapies, such as FOLFOX (fluoropyrimidine, leucovorin, and oxaliplatin)/CAPOX (capecitabine and oxaliplatin) or FOLFIRI (fluoropyrimidine, leucovorin, and irinotecan) in first- or second-line treatment of mCRC.^4^^,^^16^^,^^17^ In addition, continuous inhibition of vascular endothelial growth factor (VEGF) with BEV in second-line treatment has clinical benefits in patients with mCRC.^18^ Tsukihara et al. showed that the addition of BEV increases the antitumor effects of FTD/TPI in CRC xenografts.^19^ A phase I/II study, C-TASK FORCE, showed the promising activity of combination therapy of FTD/TPI plus BEV for mCRC refractory to standard therapies.^20^ In a subgroup analysis according to *RAS* mutation status, both PFS and OS were better in patients with *RAS* wild-type (WT) tumors than in those with *RAS* mutant (MUT) tumors, although there were no significant differences [hazard ratio (HR) in PFS: 1.755, 95% confidence interval (CI): 0.758-4.066; and HR in OS: 1.637, 95% CI: 0.674-3.980]. *RAS* mutation status is a well-known predictive marker for anti-epidermal growth factor receptor (EGFR) antibody therapy and a prognostic marker for mCRC; however, its relationship with the efficacy of FTD/TPI plus BEV is unclear.^21^, ^22^, ^23^ Owing to the small number of patients in the C-TASK FORCE trial (*N* = 25), the efficacy of this treatment according to *RAS* mutation status has not been fully clarified.

Therefore, we conducted a phase II study (JFMC51-1702-C7) to investigate the efficacy and safety of FTD/TPI plus BEV combination therapy for previously treated mCRC according to *RAS* mutation status.

## Patients and methods

### Study design

The JFMC51 study was a single-arm, two-cohort, multicenter, phase II study conducted in Japan. This study was carried out in accordance with the Declaration of Helsinki, and the protocol was approved by the Institutional Review Board of each participating center. Written informed consent was obtained from all participants. This study was registered with the Japan Registry of Clinical Trials (trial identifier: jRCTs031180104) and UMIN Clinical Trials Registry (trial identifier: UMIN000030077).

### Patient selection

The key eligibility criteria were as follows: histologically confirmed metastatic colorectal adenocarcinoma; ≥20 years of age; Eastern Cooperative Oncology Group (ECOG) performance status (PS) of 0 or 1; confirmed *RAS* mutation status using validated methods at a local laboratory; a history of one or more prior chemotherapies; refractory or intolerant to fluoropyrimidine, irinotecan, oxaliplatin, anti-VEGF therapy (BEV, ramucirumab, or aflibercept), and anti-EGFR antibody (cetuximab or panitumumab) if *RAS* WT; no history of prior FTD/TPI and regorafenib therapy; measurable disease by Response Evaluation Criteria in Solid Tumors (RECIST) version 1.1; and adequate organ function. The details of the inclusion and exclusion criteria are described in the Supplementary Material, available at https://doi.org/10.1016/j.esmoop.2021.100093 (online supplement describing patient inclusion and exclusion criteria).

### Procedures

Patients were enrolled in either the *RAS* WT or *RAS* MUT cohort according to their *RAS* mutation status. Patients were treated with FTD/TPI (35 mg/m^2^, twice daily, days 1-5 and 8-12) and BEV (5 mg/kg, intravenous infusion, days 1 and 15). The treatment course was repeated every 28 days until disease progression, unacceptable toxicity, or withdrawal of consent. If patients experienced unacceptable toxicity related to BEV, FTD/TPI monotherapy was continued according to the protocol. Administration of BEV alone was not allowed. The dose of FTD/TPI could be reduced by 10 mg/m^2^ per day until it reached a minimum dose of 40 mg/m^2^ per day according to the protocol. No reduced dose of BEV was planned in this study.

Efficacy evaluation was made according to RECIST version 1.1 every 8 weeks during the first 18 months after treatment initiation, and every 12 weeks thereafter. Adverse events were graded according to the National Cancer Institute Common Terminology Criteria for Adverse Events version 4.0, Japan Clinical Oncology Group edition every 2 weeks.

Detection of the *BRAF* V600E mutation was centrally carried out using a GENOSERCH BRAF kit (MBL, Nagoya, Japan) with the bead-based multiplex immunoassay system (xMAP Technology; Luminex).

### Endpoints

The primary endpoint was disease control rate (DCR) in both the *RAS* WT and MUT cohorts, which was assessed by the investigators. Radiologic assessment of tumors via CT scan was carried out by the investigators every 8 weeks; RECIST, version 1.1, was used to assess tumor responses. DCR was defined as complete response (CR) or partial response (PR) plus stable disease (SD) for more than 6 weeks from the initiation of treatment. Secondary endpoints were DCR for all patients, and PFS, OS, and overall response rate (ORR) for the *RAS* WT and *RAS* MUT cohorts and all eligible patients. The exploratory endpoint was to evaluate the efficacy outcomes in patients with *BRAF* V600E mutation.

### Statistical analysis

Because the difference in DCR according to *RAS* mutational status was unknown at the time of study planning, based on the results of the RECOURCE and C-TASK FORCE studies, the threshold and expected values of DCR for both *RAS* WT and *RAS* MUT cohorts were set at 44% and 65%, respectively.^10^^,^^20^ Assuming a one-sided significance level of 5.0%, the necessary sample size to achieve a power of 90% was estimated to be 49 patients (target sample size: 50 patients) in each cohort.

The efficacy analysis set was defined as all eligible patients. The safety analysis set was all treated patients. The primary analysis was an exact binomial test of DCR in each of the *RAS* WT and *RAS* MUT cohorts against the above threshold value (44%) with a one-sided significance level of 5.0%. Point estimates and two-sided 90% exact binomial CIs were computed for DCR and ORR. Waterfall plots were generated using the best percentage change in the sum of the longest diameters of measurable tumors. The Kaplan–Meier method was used to estimate the median PFS and OS with CIs calculated using the Greenwood formula. We defined PFS as the period from the date of enrollment to the date of disease progression or to the date of death, regardless of the cause of death, if the patient died without disease progression. We defined OS as the period from the date of enrollment until death due to any cause. The *RAS* mutation status and prognostic factors were analyzed using multivariable logistic regression analysis for binomial endpoints and Cox regression analysis for time-to-event endpoints, respectively. *RAS* mutation status (WT/MUT), time from diagnosis of metastasis (≥18/<18 months), sex (male/female), age (≥65/<65 years), number of prior regimens (≥3/≤2), number of metastatic sites (≥3/≤2), and location of the primary tumor [right (cecum, ascending colon, and transverse colon)/left (descending colon, sigmoid colon, and rectum)] were entered in the models. In addition, other possible covariates (ECOG PS; disease history; comorbidity; histology; and previous history of surgery, radiation therapy, and other cancer) were selected using backward variable selection (threshold exclusion criteria for *P* value = 0.20). Adjusted odds ratios (ORs) and HRs were estimated in the multivariable model. All statistical analyses were carried out using SAS version 9.4 (SAS Institute, Cary, NC).

## Results

### Patients

Between January 2018 and September 2018, 52 and 50 patients were enrolled in the *RAS* WT and *RAS* MUT cohorts, respectively, from 34 centers across Japan (Supplementary Figure S1, available at https://doi.org/10.1016/j.esmoop.2021.100093). All 102 patients received FTD/TPI plus BEV combination therapy; 5 patients were ineligible, including 3 patients who enrolled in this study within 2 weeks of completing a previous therapy, 1 patient who had no prior history of anti-VEGF therapy, and 1 patient who had another type of active cancer.

Baseline characteristics in the efficacy analysis set in each cohort are summarized in Table 1. In the *RAS* MUT cohort, ECOG PS1 and right-sided primary tumor were frequently enrolled. There were significant differences in the number of prior therapies and the time to enroll in this study between the *RAS* WT and *RAS* MUT cohorts. *BRAF* V600E mutation was detected in the *RAS* WT cohort (five patients, 10%).

**Table 1:** Baseline characteristics of patients

Characteristics	*RAS* wild-type *n* = 48 (%)	*RAS* mutant *n* = 49 (%)	*P* value
Age, years			
Median (range)	65 (33-85)	64 (37-82)	0.6754
>65	25 (52)	24 (49)	0.7598
<65	23 (48)	25 (51)	
Sex			
Male	24 (50)	29 (59)	0.3634
Female	24 (50)	20 (41)	
ECOG PS			
0	33 (69)	29 (59)	0.3266
1	15 (31)	20 (41)	
Location of primary tumor			
Right	8 (17)	16 (33)	0.0681
Left	40 (83)	33 (67)	
Adjuvant therapy			
Yes	48 (100)	49 (100)	
No	0 (0)	0 (0)	
Metastatic site			
≤2	30 (63)	34 (69)	0.4741
≥3	18 (38)	15 (31)	
Site of metastasis			
Liver	33 (69)	36 (73)	0.6081
Lung	29 (60)	32 (65)	0.6182
Lymph nodes	21 (44)	16 (33)	0.2606
Peritoneum	11 (23)	15 (31)	0.3923
Bone	4 (8)	2 (4)	0.3848
*BRAF*			
Wild-type	41 (85)	46 (94)	0.0646
Mutant	5 (10)	0 (0)	
Unknown	2 (4)	3 (6)	
Number of prior regimens			
≤2	5 (10)	21 (43)	0.0003
≥3	43 (90)	28 (57)	
Time from diagnosis of metastasis			
<18 months	7 (15)	18 (37)	0.0126
≥18 months	41 (85)	31 (63)	

ECOG, Eastern Cooperative Oncology Group; PS, performance status.

### Treatment

The data cut-off date for this analysis was 16 December 2019, and the median follow-up was 15.8 months (15.2 months in the *RAS* WT cohort and 16.1 months in the *RAS* MUT cohort). The median numbers of treatment courses were four courses (range: 1-10) in the *RAS* WT cohort and three courses (range: 1-10) in the *RAS* MUT cohort (Supplementary Table S1, available at https://doi.org/10.1016/j.esmoop.2021.100093). A dose reduction of FTD/TPI was required in 23% and 16% of patients in the *RAS* WT and *RAS* MUT cohorts, respectively. Approximately 60% of patients in each cohort had delays in starting the subsequent course. The most common reason for the dose reduction and delay was treatment-related neutropenia. The median relative dose intensities (RDIs) of FTD/TPI were 88% and 84%, and the mean RDIs of FTD/TPI were 87% and 83% in the *RAS* WT and *RAS* MUT cohorts, respectively. The median RDIs of BEV were 89% and 81%, and the mean RDIs of BEV were 86% and 82% in the *RAS* WT and *RAS* MUT cohorts, respectively.

### Efficacy

The DCRs for the *RAS* WT and *RAS* MUT cohorts were 66.7 (90% CI: 53.9%-77.8%, *P* = 0.0013) and 55.1% (90% CI: 42.4%-67.3%, *P* = 0.0780), respectively (unadjusted OR, 0.61; 90% CI, 0.31-1.22; Table 2 and Supplementary Table S2, available at https://doi.org/10.1016/j.esmoop.2021.100093).

**Table 2:** Response to treatment

	*RAS* wild-type *n* = 48	*RAS* mutant *n* = 49	All *N* = 97
Disease control rate	66.7%	55.1%	60.8%
90% (CI)	(53.9-77.8)	(42.4-67.3)	(52.0-69.2)
*P* value	0.0013	0.0780	—
Objective response rate	6.3%	0%	3.1%
90% (CI)	(1.7-15.4)	—	(0.8-7.8)
Best overall response			
Complete response	0	0	0
Partial response	3	0	3
Stable disease	29	27	56
Progressive disease	15	22	37
Not evaluated	1	0	1

CI, confidence interval.

**Figure 1:**
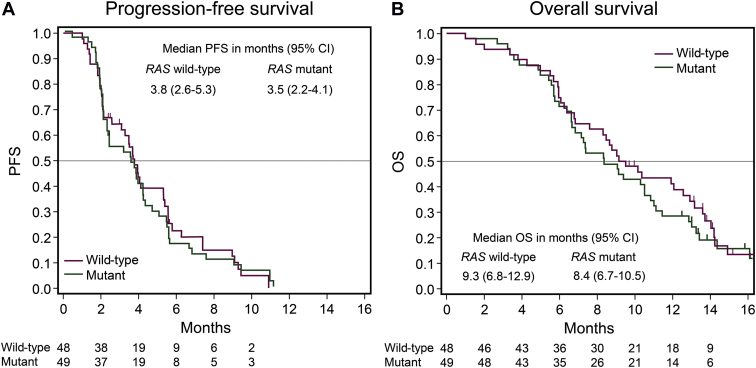
Progression-free survival and overall survival according to *RAS* mutation status. Kaplan–Meier estimates of progression-free survival (A) and overall survival (B). CI, confidence interval; OS, overall survival; PFS, progression-free survival.

The lower limit of the 90% CI in the *RAS* WT cohort was higher than the prespecified threshold of 44%, whereas that in the *RAS* MUT cohort was not. No patients achieved CR in both cohorts, and PR was observed only in the *RAS* WT cohort (three patients), resulting in an ORR of 6.3% (90% CI: 1.7-15.4). Additionally, 41.3% of patients in the *RAS* WT cohort and 26.5% of patients in the *RAS* MUT cohort experienced tumor shrinkage (Supplementary Figure S2, available at https://doi.org/10.1016/j.esmoop.2021.100093). The median PFS in the *RAS* WT and *RAS* MUT cohorts were 3.8 (95% CI: 2.6-5.3) and 3.5 months (95% CI: 2.2-4.1), respectively (HR: 1.14, 95% CI: 0.76-1.73; Figure 1A). The median OS in the *RAS* WT and *RAS* MUT cohorts was 9.3 (95% CI: 6.8-12.9) and 8.4 months (95% CI: 6.7-10.5), respectively (HR: 1.15, 95% CI: 0.74-1.78; Figure 1B). Univariate and multivariate regression analyses showed better trends in DCR, PFS, and OS in the *RAS* WT cohort than in the *RAS* MUT cohort; however, there were no significant differences [adjusted OR: 0.48 (90% CI: 0.22-1.08) in DCR; adjusted HR: 1.56 (95% CI: 0.94-2.60) in PFS; and adjusted HR: 1.29 (95% CI: 0.76-2.20) in OS; Supplementary Table S2, available at https://doi.org/10.1016/j.esmoop.2021.100093 (univariate) and Table 3 (multivariate)]. There were no other statistically significant factors, except for the location of the primary tumor on PFS in multivariate analysis.

**Table 3:** Multivariable regression analysis of DCR, PFS, and OS

Variables	Factor	*n*	DCR			PFS			OS		
			OR	90% CI	*P* value	HR	95% CI	*P* value	HR	95% CI	*P* value
*RAS* mutation status (mutant/wild-type)	Mutant Wild-type	49 48	0.48	0.22-1.08	0.1362	1.56	0.94-2.60	0.0870	1.29	0.76-2.20	0.3404
Time from diagnosis of metastasis (≥18/<18 months)	≥18 <18	72 25	1.06	0.43-2.65	0.9107	0.89	0.52-1.52	0.6577	0.89	0.50-1.58	0.6862
Sex (female/male)	Female Male	44 53	1.20	0.56-2.57	0.6865	1.14	0.72-1.80	0.5770	1.11	0.69-1.80	0.6649
Age, years (≥65/<65)	≥65 <65	49 48	0.68	0.32-1.42	0.3912	1.16	0.73-1.83	0.5325	0.99	0.61-1.60	0.9503
Number of prior regimens (≥3/≤2)	≥3 ≤2	71 26	0.68	0.26-1.73	0.4959	1.38	0.79-2.41	0.2648	0.95	0.51-1.77	0.8795
Number of metastatic sites (≥3/≤2)	≥3 ≤2	33 64	0.74	0.35-1.57	0.5084	1.55	0.98-2.46	0.0595	1.59	0.99-2.56	0.0550
Location of primary tumor (right/left)	Right Left	24 73	1.82	0.73-4.57	0.2814	0.53	0.29-0.98	0.0440	0.63	0.33-1.21	0.1650

CI, confidence interval; DCR, disease control rate; HR, hazard ratio; OR, odds ratio; OS, overall survival; PFS, progression-free survival.

In all patients in the efficacy analysis set, DCR, median PFS, and OS were 60.8% (90% CI: 52.0-69.2), 3.7 months (95% CI: 2.6-4.1), and 9.1 months (95% CI: 7.4-10.5), respectively (Table 2, Supplementary Figure S3, available at https://doi.org/10.1016/j.esmoop.2021.100093).

Among the five patients with the *BRAF* V600E mutation, no patients achieved PR; three patients showed SD, and two patients showed PD. The median PFS and OS in patients with the *BRAF* V600E mutation were 3.5 months (95% CI: 1.1-10.9) and 8.5 months (95% CI: 3.4-13.7), respectively (Supplementary Figure S4, available at https://doi.org/10.1016/j.esmoop.2021.100093).

### Safety

No statistically significant differences were observed in the incidence of adverse events between the *RAS* WT and *RAS* MUT cohorts. The major grade 3 or higher adverse events in the *RAS* WT and *RAS* MUT cohorts were neutropenia (46% and 62%, respectively), anemia (10% and 22%, respectively), anorexia (12% and 10%, respectively), hypertension (15% and 18%, respectively), and protein urea (13% and 4%, respectively; Table 4).

**Table 4:** Adverse events

Adverse event	*RAS* wild-type *n* = 52		*RAS* mutant *n* = 50		*P* value	All *N* = 102	
	Any (%)	Grade >3 (%)	Any (%)	Grade >3 (%)		Any (%)	Grade >3 (%)
Neutropenia	71	46	82	62	0.1085	76	54
Thrombocytopenia	56	6	58	8	0.6560	57	7
Anemia	81	10	88	22	0.0856	84	16
Febrile neutropenia	6	6	2	2	0.3269	4	4
Nausea	65	2	52	8	0.1553	59	5
Anorexia	87	12	72	10	0.8023	79	11
Diarrhea	35	8	38	2	0.1832	36	5
Fatigue	79	2	78	0	0.3244	78	1
Hypertension	67	15	78	18	0.7231	73	17
Proteinuria	75	13	72	4	0.0921	74	9

Although grade 3 or higher neutropenia was the most common adverse event in all patients defined as the safety analysis set (54%), the incidence of febrile neutropenia was 4%. During the treatment, one patient in the *RAS* WT cohort, a 74-year-old male with diabetes, died of myocardial infarction.

### Subsequent chemotherapy

At the data cut-off date, the protocol treatment had been discontinued in all patients defined as the efficacy analysis set. The reasons for discontinuation were disease progression (88%), adverse events (9%), patient refusal (1%), and others (2%). In the *RAS* WT and *RAS* MUT cohorts, 75% and 67% of patients received subsequent chemotherapy, and 40% and 47% of patients were treated with regorafenib, respectively (Supplementary Table S3, available at https://doi.org/10.1016/j.esmoop.2021.100093).

## Discussion

To the best of our knowledge, this is the first study to prospectively analyze the efficacy and safety of FTD/TPI plus BEV therapy according to *RAS* mutation status. The primary endpoint, DCR, was significantly higher than the prespecified threshold value in the *RAS* WT cohort, but not in the *RAS* MUT cohort. Since the DCR threshold and expected value of the primary endpoint were set based on previous results (C-TASK FORCE, J-003, and RECOURSE), the DCR in this study was defined in the same manner it was in the above studies.^10^^,^^20^^,^^24^ The multivariable analysis also showed better trends in all efficacy outcomes in the *RAS* WT cohort than the *RAS* MUT cohort, although there were no significant differences. This result could be interpreted in two ways: first, *RAS* mutation status may be a predictive factor for the efficacy of FTD/TPI plus BEV, or second, this difference may be a reflection of the poor prognosis of the *RAS* MUT, as reported previously.^11^ A recently published randomized phase II study, the Danish trial, compared FTD/TPI plus BEV with FTD/TPI for patients with previously treated mCRC; the results showed that the addition of BEV to FTD/TPI significantly increased PFS and OS, irrespective of *RAS* mutation status.^25^ In addition, the DCR in this study was better than that in FTD/TPI monotherapy previously reported, even in the *RAS* MUT cohort (55.1% versus 43%-44%).^10^^,^^11^^,^^24^ Taking these factors into account, we interpreted our results to indicate that the combination of FTD/TPI plus BEV could improve the efficacy outcomes of previously treated mCRC, regardless of *RAS* mutation status, although patients with the *RAS* MUT showed relatively poor prognoses.

In the multivariable analysis in this study, a statistical significance was observed in PFS only for the location of the primary tumor (HR: 0.53, 95% CI: 0.20-0.98, *P* = 0.0440), and PFS was better in the right-sided tumor than the left-sided tumor. A subgroup analysis of the randomized phase III study, NCIC CO.17 trial, which compared cetuximab with the best supportive care for patients with previously treated mCRC, showed that the location of the primary tumor in the best supportive care group is not prognostic for OS or PFS (HR in OS: 0.96, HR in PFS: 1.07).^26^ In the subgroup analysis of the Danish trial, the effects of adding BEV to FTD/TPI on PFS were better in the right-sided tumor than the left-sided tumor (HR in right-sided tumor: 0.37, HR in the left-sided tumor: 0.49).^25^ Based on these results, the location of the primary tumor may be a predictive factor for the efficacy of FTD/TPI plus BEV, regardless of *RAS* mutation status; however, because of the small number of patients with right-sided tumors (24 patients in this study and 22 patients in the Danish trial), additional studies are still needed.

*BRAF* V600E mutation is recognized as a strong prognostic factor, with an impressive negative impact on mCRC survival.^27^ In this study, we found similar results in DCR, PFS, and OS between patients with *BRAF* V600E mutation and wild-type *BRAF*, and one patient with *BRAF* V600E mutation received FTD/TPI plus BEV for approximately 1 year. The *BRAF* V600E mutation is commonly detected in patients with MSI-high tumors, and FTD/TPI has been shown to enhance the antitumor activity against MSI-high tumors in a preclinical study and the C-TASK FORCE trial.^20^^,^^28^, ^29^, ^30^, ^31^ Although the number of patients with *BRAF* V600E mutation was small, and MSI testing was not carried out in this study, no impact of *BRAF* V600E mutation status on the efficacy of FTD/TPI plus BEV was suggested.

There were no new findings regarding the safety profiles of FTD/TPI plus BEV in this study. Grade 3 or higher neutropenia was the most common adverse event and more frequently observed in FTD/TPI plus BEV than FTD/TPI monotherapy, as reported in the C-TASK FORCE trial and Danish trial; however, the incidence of febrile neutropenia was low, and this adverse event is considered manageable.^20^^,^^25^ Some differences in the incidence of adverse events between *RAS* mutation status were observed; grade 3 or higher neutropenia and anemia were frequently observed in the *RAS* MUT cohort. This difference may have been influenced by patient characteristics, particularly ECOG PS1, which was more frequent in the *RAS* MUT cohort.

This study had several limitations. First, the number of patients was small. However, this is the largest study to evaluate the efficacy and safety of FTD/TPI plus BEV according to *RAS* mutation status among three phase II studies in late-line treatment (*RAS* WT/MUT: 48/49 patients in this study, 10/15 patients in C-TASK FORCE trial, 19/27 patients in Danish trial).^20^^,^^25^ For the analysis of primary tumor location and *BRAF* V600E mutation, further studies in larger cohorts are warranted. Second, this study had a single-arm design, and we could not validate the efficacy and safety of FTD/TPI plus BEV over FTD/TPI monotherapy or the predictive impact of *RAS* mutational status and location of the primary tumor. However, a randomized phase II study, the Danish trial, showed promising efficacy outcomes with tolerable safety profiles for FTD/TPI plus BEV compared with FTD/TPI, and our results were consistent with the results of the Danish trial.^25^ Thus, we believe that the current findings were clinically valuable. The current on-going phase III studies, the SOLSTICE trial, investigating capecitabine plus BEV in first-line treatment, and the TRUSTY trial investigating FOLFIRI plus BEV in second-line treatment, will provide solid information of FTD/TPI plus BEV.

In conclusion, the combination therapy of FTD/TPI plus BEV showed promising activity with an acceptable safety profile for previously treated mCRC, regardless of *RAS* mutation status, although the efficacy outcomes tended to be better in *RAS* WT.
