# Supplementary material for: Phase II study of trifluridine/tipiracil plus bevacizumab by RAS mutation status in patients with metastatic colorectal cancer refractory to standard therapies: JFMC51-1702-C7
Source: ESMO Open. 2021 Mar 19;6(2):100093. doi: 10.1016/j.esmoop.2021.100093 (PMC7985393; doi:10.1016/j.esmoop.2021.100093)
Supplement: Supplementary Material [file mmc1.docx]

**Supplementary Materials**

**Patient inclusion and exclusion criteria**

Inclusion criteria:

1. Histologically confirmed advanced or recurrent colorectal adenocarcinoma
2. Unresectable disease confirmed using CT or MRI scans
3. ECOG performance status of 0–1
4. Confirmed *RAS* mutation status via validated methods at the local laboratory
5. Age > 20 years
6. History of at least 1 standard chemotherapy for unresectable colorectal cancer and failure or intolerant to this therapy
   1. Standard chemotherapy must include all of the following drugs:
      1. Fluoropyrimidine, Irinotecan, and Oxaliplatin
      2. Bevacizumab, Ramucirumab, or Aflibercept
      3. Anti-EGFR antibody (Cetuximab or Panitumumab) if *RAS* wild-type
   2. Patients who discontinued standard chemotherapy before progression owing to intolerance to the toxicity of drugs except for bevacizumab are eligible
   3. If relapsed within 6 months after the last dose of adjuvant chemotherapy, this adjuvant chemotherapy is considered as the 1^st^-line chemotherapy
7. No treatment history of Regorafenib and FTD/TPI
8. Can take oral medication
9. Measurable disease using CT or MRI scans performed within 21 days before registration per RECIST 1.1 criteria
10. Adequate organ function (data obtained within 14 days before registration)
    1. Hemoglobin (Hb) > 9.0 g/dL
    2. Neutrophil count ≥ 1,500 /mm^3^
    3. Platelet count ≥ 100,000 /mm
    4. Total bilirubin ≤ 1.5 mg/dL
    5. AST/ ALT < 100 IU/L

(both AST and ALT levels < 200 IU/L in patients with liver metastasis)

- 1. Serum creatinine < 1.5 mg/dL
  2. Proteinuria with at least one of the following:
     1. Urine dipstick < 1+
     2. Urine protein/creatinine ratio < 1.0
     3. 24-h urine < 1 g/24 h

1. Treatment can be started within 14 days after registration
2. Willingness to comply with scheduled visits and protocol treatment procedures
3. Written informed consent with the patient’s signature and date

Exclusion criteria:

1. Contraindications for bevacizumab
2. Complications or history of thromboembolism and severe lung disease (interstitial pneumonia, pulmonary fibrosis, severe emphysema, etc.) within 6 months before registration, except for central venous catheter-related thrombosis that does not currently require treatment
3. Active bleeding
4. Myocardial infarction, severe unstable angina, or symptomatic congestive heart failure (NYHA Class III/IV) within 12 months before registration
5. Cerebrovascular disease
6. Active infection (patients with fever of at least 38 ℃ owing to infection)
7. Body cavity fluid (pleural, ascitic, and pericardial fluid) requiring treatment
8. Intestinal obstruction, renal failure, or liver failure
9. Uncontrolled diabetes mellitus
10. Uncontrolled hypertension
11. Cirrhosis, HBs antigen-positive, or HCV antibody-positive
12. Other active malignancies less than 5 years free of disease, except for intraepithelial (mucosal) cancer considered cured
13. Brain or meningeal metastases
14. History of autoimmune deficiency or organ transplantation requiring immunosuppressive therapy
15. Received any of the following treatments within a certain period before registration:
    1. Thoracotomy or laparotomy within the last 4 weeks
    2. Any anti-cancer drug treatment within the last 2 weeks
    3. Irradiation of a wide range (more than 30% of hematopoietic bone marrow) within the last 4 weeks or irradiation of a limited range within the last 2 weeks
16. Remaining grade 2 or higher adverse events owing to prior therapy, except for anemia, alopecia, skin pigmentation, oxaliplatin-related peripheral neuropathy, angiogenesis inhibitor-related hypertension, or anti-EGFR antibody-related hypomagnesemia and hypocalcemia
17. Administration of blood transfusions or hematopoietic factor preparations within 14 days before registration
18. An unhealed wound or traumatic fracture
19. Bleeding tendency or taking antithrombotic drugs such as aspirin over 325 mg/day
20. Pregnant women, lactating women, women with a positive pregnancy test, or men wishing to have a partner's pregnancy
21. Clinically significant mental or psychological disorder
22. Considered inappropriate for this study by a physician

**Supplementary Table S1. Treatment exposure**

|  | *RAS* wild-type  N = 48 | *RAS* mutant  N = 49 | All  N = 97 |
| --- | --- | --- | --- |
| Course, median (range) | 4 (1-10) | 3 (1-10) | 3 (1-10) |
| FTD/TPI |  |  |  |
| Dose reduction | 23% | 16% | 20% |
| Interruption | 17% | 16% | 16% |
| Delay | 54% | 69% | 62% |
| RDI | 88% (48-100) | 84% (30-100) | 86% (30-100) |
| BEV |  |  |  |
| Skip | 19% | 18% | 19% |
| Delay | 63% | 65% | 64% |
| RDI | 89% (50-100) | 81% (50-100) | 83% (50-100) |

FTD/TPI, trifluridine/tipiracil; BEV, Bevacizumab; RDI, relative dose intensity

**Supplementary Table S2. Univariate regression analysis of DCR, PFS, and OS**

| Variables | Factor | N | DCR (%) | OR  90% CI | PFS (Month) | HR  95% CI | OS (Month) | HR,  95% CI |
| --- | --- | --- | --- | --- | --- | --- | --- | --- |
| *RAS* mutation status  (mutant / wild-type) | Mutant | 49 | 27 (55%) | 0.61 | 3.52 | 1.14 | 8.38 | 1.15 |
|  | Wild-type | 48 | 32 (67%) | [0.31,1.22] | 3.78 | [0.76,1.73] | 9.35 | [0.74,1.78] |
| Time from diagnosis of metastasis  (≥18 / <18 months) | ≥ 18 | 72 | 44 (61%) | 1.05 | 3.71 | 1.01 | 9.10 | 0.88 |
|  | < 18 | 25 | 15 (60%) | [0.48,2.29] | 3.52 | [0.63, 1.61] | 8.48 | [0.54,1.46] |
| Sex  (Female / Male) | Female | 44 | 29 (66%) | 1.48 | 3.71 | 0.96 | 9.05 | 1.03 |
|  | Male | 53 | 30 (57%) | [0.74,2.97] | 3.48 | [0.64,1.46] | 9.13 | [0.66,1.59] |
| Age  (≥65 / <65) | ≥ 65 | 49 | 28 (57%) | 0.73 | 3.52 | 0.98 | 9.07 | 0.80 |
|  | < 65 | 48 | 31 (65%) | [0.37,1.45] | 3.78 | [0.65,1.48] | 9.08 | [0.51,1.24] |
| Number of prior regimens  (≥3 / ≤2) | ≥ 3 | 71 | 43 (61%) | 0.96 | 3.52 | 1.16 | 9.17 | 0.90 |
|  | ≤ 2 | 26 | 16 (62%) | [0.44,2.08] | 3.79 | [0.72,1.86] | 8.36 | [0.55,1.49] |
| Number of metastatic sites  (≥3 / ≤2) | ≥ 3 | 33 | 19 (58%) | 0.81 | 3.78 | 1.46 | 8.31 | 1.48 |
|  | ≤ 2 | 64 | 40 (63%) | [0.40,1.67] | 3.68 | [0.93,2.29] | 10.51 | [0.94,2.33] |
| Location of the primary tumor  (Right / Left) | Right | 24 | 16 (67%) | 1.40 | 3.98 | 0.76 | 10.41 | 0.77 |
|  | Left | 73 | 43 (59%) | [0.62,3.14] | 3.68 | [0.47,1.24] | 8.77 | [0.45,1.30] |

DCR, disease control rate; PFS, progression-free survival; OS, overall survival; OR, odds ratio; HR, hazard ratio; CI, confidence interval

**Supplementary Table S3. Subsequent chemotherapy**

|  | *RAS* wild-type  N = 48 | *RAS* mutant  N = 49 | All  N = 97 |
| --- | --- | --- | --- |
| Subsequent chemotherapy |  |  |  |
| No | 12 | 16 | 28 |
| Yes | 36 | 33 | 69 |
| Regorafenib | 40% | 47% | 43% |
| FTD/TPI | 17% | 22% | 20% |
| Fluoropyrimidine | 21% | 18% | 20% |
| Irinotecan | 19% | 12% | 15% |
| Oxaliplatin | 10% | 8% | 9% |
| Angiogenesis inhibitor | 25% | 35% | 30% |
| Anti-EGFR | 23% | 0% | 11% |
| Others | 2% | 2% | 2% |

FTD/TPI, trifluridine/tipiracil

**
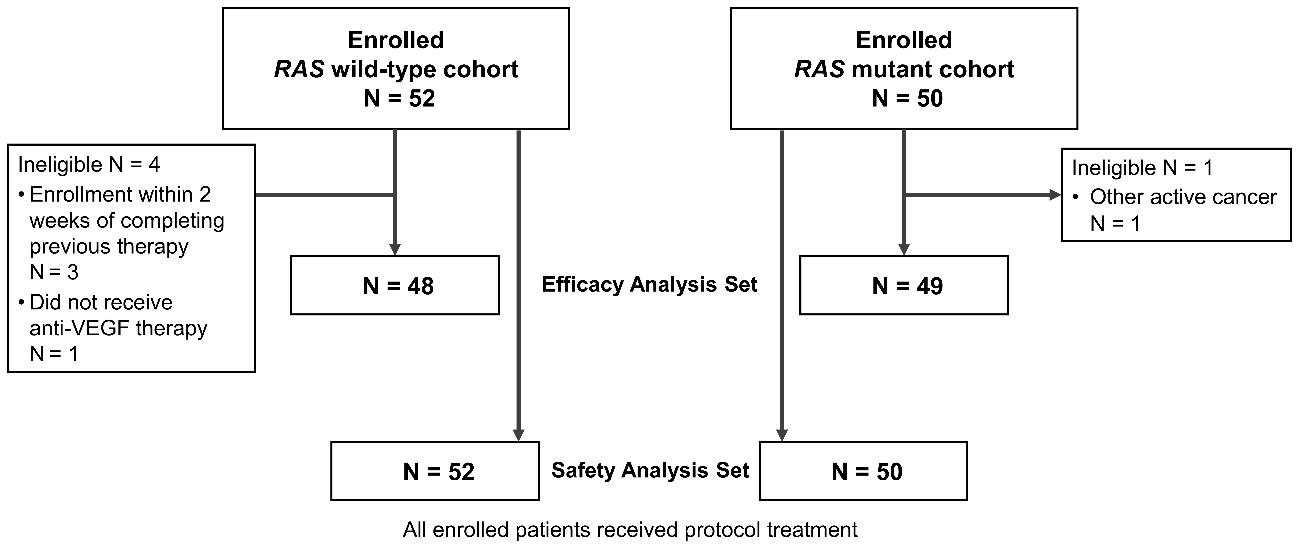
**

**Supplementary Figure S1. Trial profile.**

**
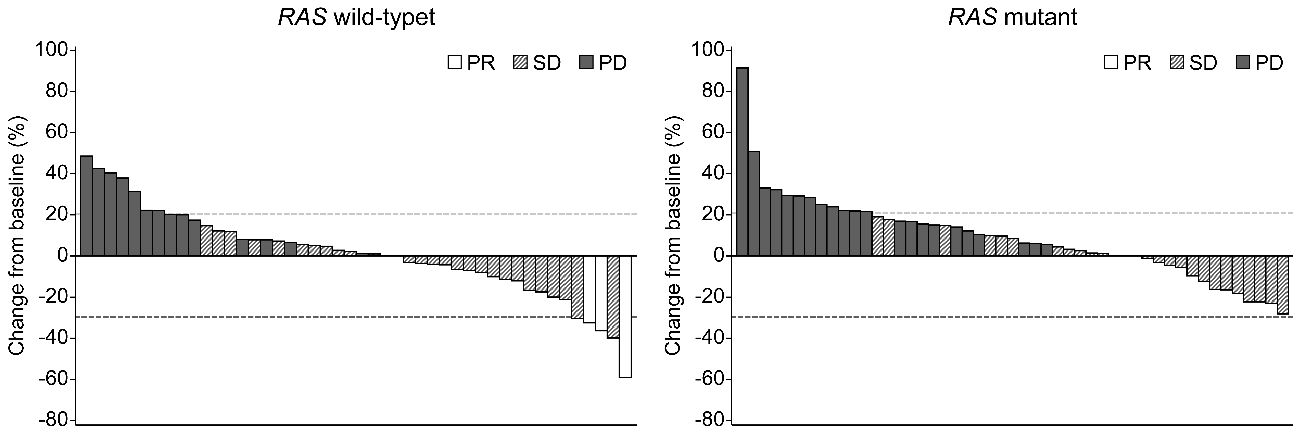
**

**Supplementary Figure S2. Waterfall plot.** PR, partial response; SD, stable disease, PD, progressive disease


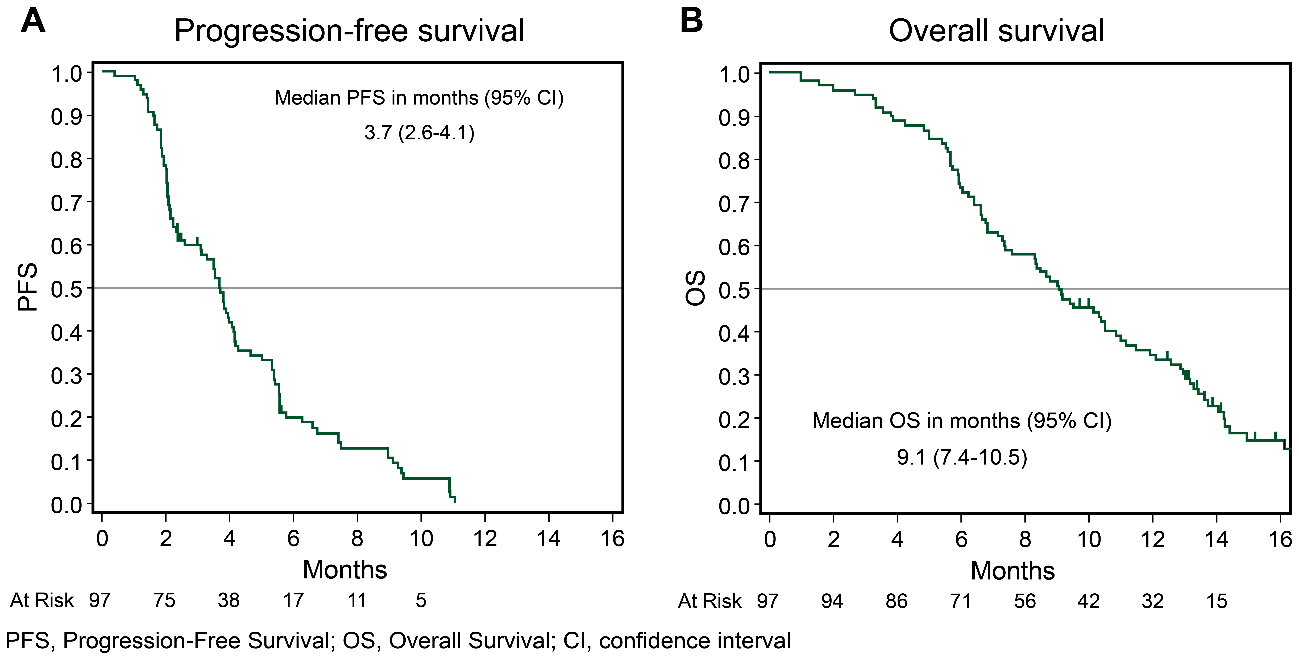


**Supplementary Figure S3. Progression-free survival and overall survival in all patients**


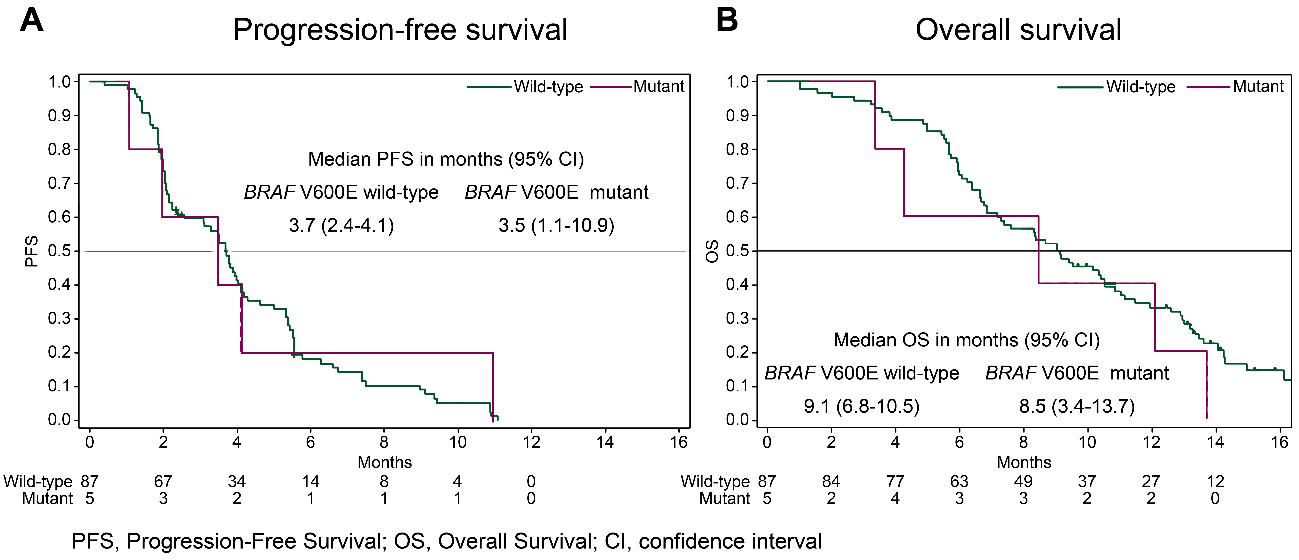


**Supplementary Figure S4. Progression-free survival and overall survival according to *BRAF* V600E mutation status.** Kaplan-Meier estimates of progression-free survival (A) and overall survival (B). PFS, progression-free survival; OS, overall survival; CI, confidence interval
